# Analyzing student mental health with RoBERTa-Large: a sentiment analysis and data analytics approach

**DOI:** 10.3389/fdata.2025.1615788

**Published:** 2025-10-17

**Authors:** Hikmat Ullah Khan, Anam Naz, Fawaz Khaled Alarfaj, Naif Almusallam

**Affiliations:** ^1^Department of Information Technology, University of Sargodha, Sargodha, Pakistan; ^2^Department of Management Information Systems, School of Business, King Faisal University, Al Ahsa, Saudi Arabia

**Keywords:** large language model, mental health, academic performance, natural language processing, sentiment analysis

## Abstract

The mental health of students plays an important role in their overall wellbeing and academic performance. Growing pressure from academics, co-curricular activities such as sports and personal challenges highlight the need for modern methods of monitoring mental health. Traditional approaches, such as self-reported surveys and psychological evaluations, can be time-consuming and subject to bias. With advancement in artificial intelligence (AI), particularly in natural language processing (NLP), sentiment analysis has emerged as an effective technique for identifying mental health patterns in textual data. However, analyzing students' mental health remains a challenging task due to the intensity of emotional expressions, linguistic variations, and context-dependent sentiments. In this study, our primary objective was to investigate the mental health of students by conducting sentiment analysis using advanced deep learning models. To accomplish this task, state-of-the-art Large Language Model (LLM) approaches, such as RoBERTa (a robustly optimized BERT approach), RoBERTa-Large, and ELECTRA, were used for empirical analysis. RoBERTa-Large, an expanded architecture derived from Google's BERT, captures complex patterns and performs more effectively on various NLP tasks. Among the applied algorithms, RoBERTa-Large achieved the highest accuracy of 97%, while ELECTRA yielded 91% accuracy on a multi-classification task with seven diverse mental health status labels. These results demonstrate the potential of LLM-based approaches for predicting students' mental health, particularly in relation to the effects of academic and physical activities.

## 1 Introduction

Advancements in AI have significantly improved the ability to process vast volumes of textual data, enabling the interpretation of user interactions to extract meaningful insights (Chancellor and De Choudhury, 2020). One of the most impactful applications of AI is in sentiment analysis, where NLP techniques are used to assess emotions, opinions, and mental states (Ishfaq et al., 2025). With the rise of social media and digital platforms, individuals frequently express their thoughts, feelings, and experiences through comments, posts, and reviews (Uban et al., 2021). This user-generated content (UGC) serves as a valuable resource for understanding public sentiment regarding mental health (Primack et al., 2018). Mental health is a crucial aspect of overall wellbeing, significantly influencing an individual's emotional stability, productivity, and quality of life (Ahmad et al., 2023). However, analyzing mental health trends based on online sentiment is a challenging task due to the complexity of human emotions, context-dependent language, and diverse expressions of psychological distress (Aragón et al., 2023). Social media often reflects a wide spectrum of sentiments, ranging from positive encouragement to severe distress signals related to depression, anxiety, and suicidal thoughts (Roemmich and Andalibi, 2021). Detecting such emotions accurately requires sophisticated AI-driven models capable of identifying nuanced linguistic patterns and contextual meanings (Alsini et al., 2024; Li et al., 2025).

**Figure 1:**
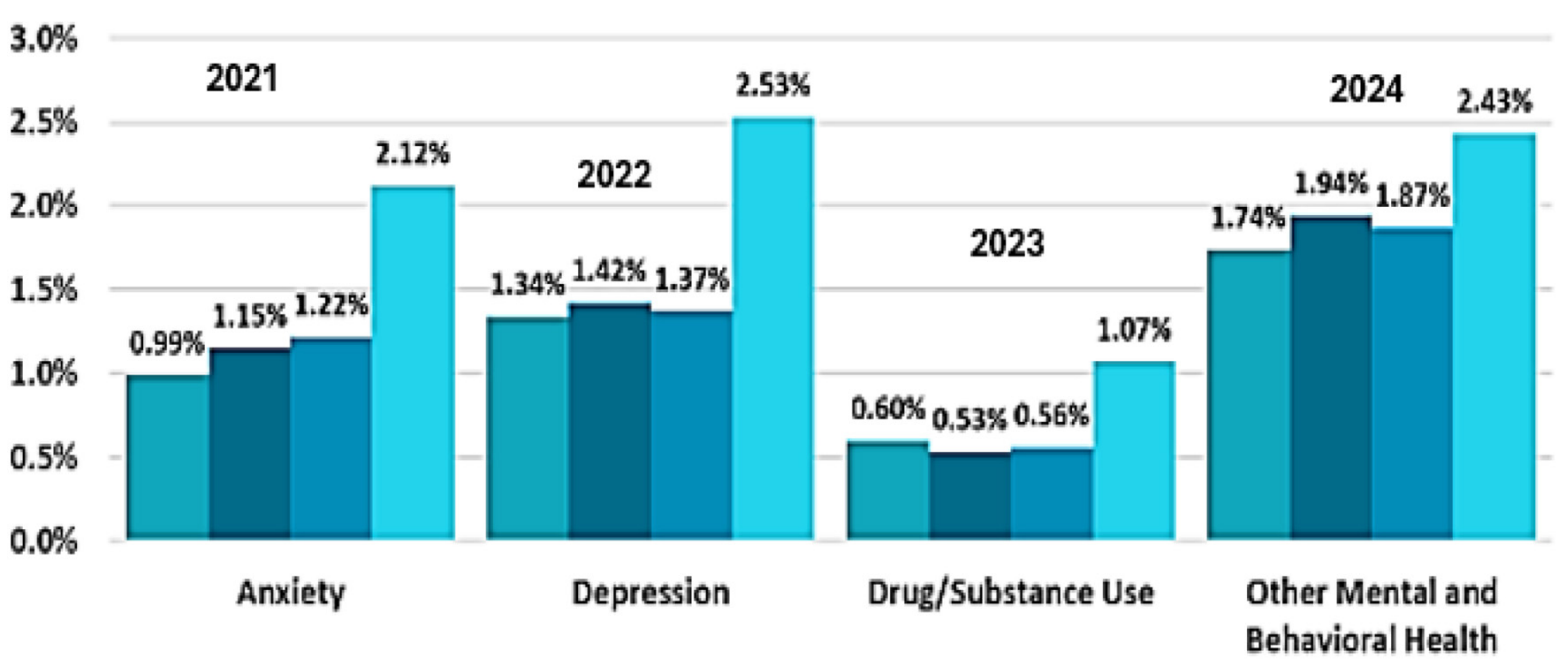
Trend analysis of rise in mental health states.

The increasing rate of mental health disorders highlights the importance of AI-driven sentiment analysis. According to studies, the cases of depression and anxiety have significantly increased in recent years, with statistics in the post-pandemic scenario showing a rise of 25% in mental health issues. As also shown in Figure 1, a distressing trend is the increase in suicides, with >700,000 suicides occurring per year around the world (Saraceno and Caldas De Almeida, 2022). Sentiment analysis on a large scale of UGC can be used to help researchers identify early warning signs, track mental health trends, and develop targeted interventions for coping with psychological distress efficiently (Ding et al., 2023). Sentiment classification has advanced to a more sophisticated level through the use of deep learning models, such as BERT, GPT, and transformer-based architectures. These models utilize contextual embedding's and attention to identify complex emotional cues in the text data. Adopting an AI-based strategy may help in early detection systems working in mental health and psychology, therapy, and policymaking. Furthermore, categorizing mental health discussions by risk level can help provide people with timely and tailored support (Babu and Kanaga, 2021).

Sentiment analysis powered by deep learning offers a promising approach to understanding mental health trends in the digital era. With the advancement of AI, it is now possible to utilize AI to bridge the gap between early diagnosis and intervention in mental health research (Zhu, 2023). In this study, our primary objective is to predict mental health states using sentiment analysis from UGC. For empirical analysis, a self-prepared dataset has been used. For feature extraction and prediction of mental health status, we utilized state-of-the-art transformer-based and baseline models.

The main contributions of this study to sentiment analysis in mental health using deep learning techniques are as follows:

- Application of modern transformer models, namely RoBERTa-Large and ELECTRA, in classifying UGC into various mental health statuses. The results show that RoBERTa-Large achieves 97% accuracy, outperforming ELECTRA with 91% accuracy, which highlights the feasibility of using contextual embedding's in sentiment classification.
- Explored the effect of working with mental health trends by integrating deep learning with NLP. This research leveraged the analysis of sentiment in social media discussions to gain insights into mental health conditions such as depression, anxiety, and suicidal tendencies, aiming to develop a data-driven understanding of mental wellbeing.
- Developed a robust framework for AI-based mental health monitoring using sentiment analysis, which can be used in mental health support systems to help people by providing timely recommendations based on the sentiment patterns found in UGC.

**Figure 2:**
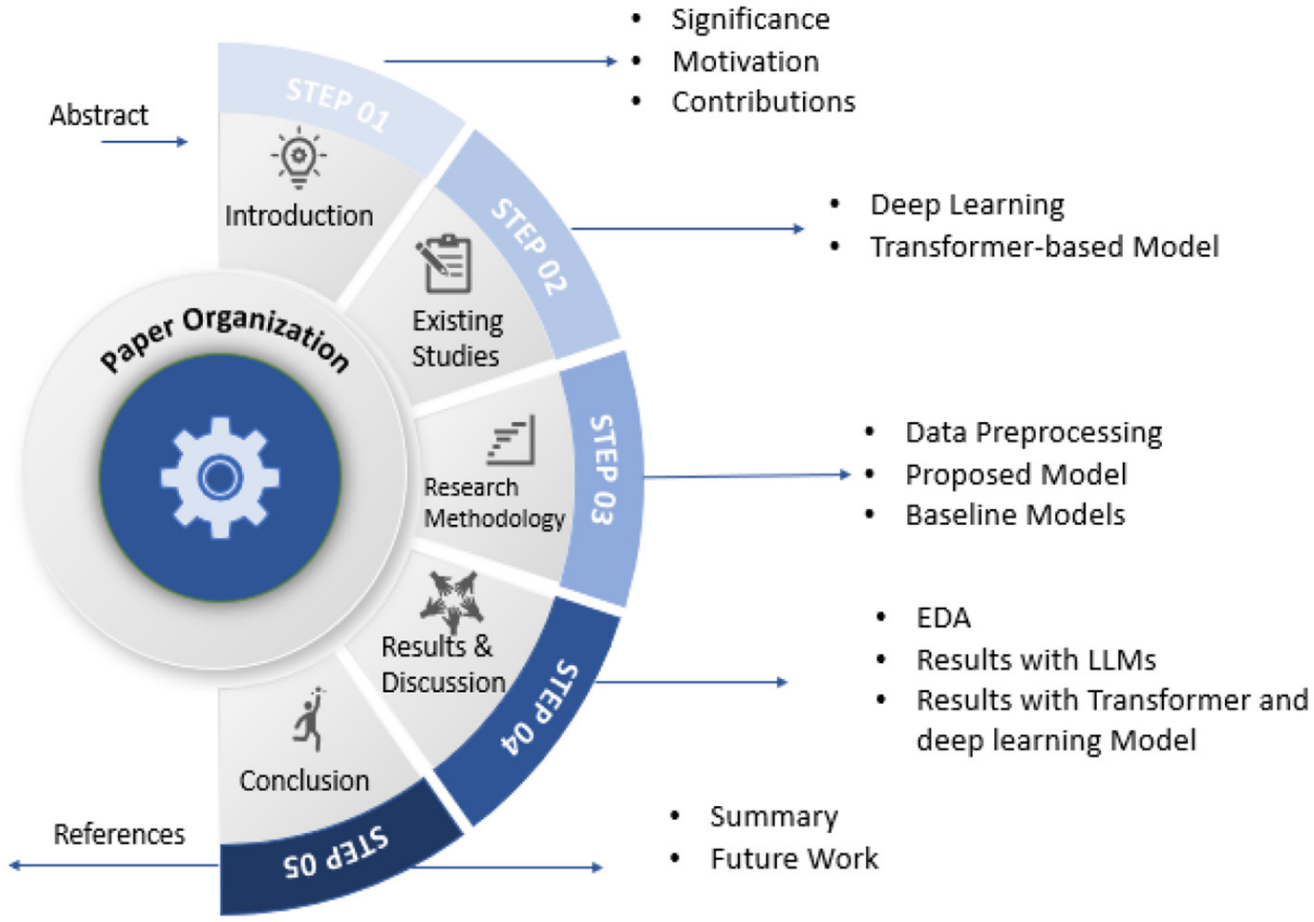
Organization of paper.

The remainder of the paper, as outlined in Figure 2, is organized as follows: Section 2 presents a comprehensive analysis of the existing literature, with a focus on deep learning techniques. Section 3 provides the roadmap of this study by discussing the steps of the proposed methodology. Section 4 presents a comprehensive analysis of the results, along with a detailed discussion. Section 5 summarizes the study by presenting conclusions and outlining future directions.

## 2 Related research

Research over the past few years has demonstrated how deep learning and transformer-based models effectively utilize textual data to predict mental health outcomes. Multiple research projects have applied these sophisticated approaches to sentiment evaluation and mental healthcare forecasts because they show substantial promise for spotting depression, along with anxiety and suicidal risk factors. Within current research on mental health prediction, the main challenges that persist include coping with linguistic diversity in expressions, managing ethical issues, and the need to combine multiple data types. Table 1 presents an analysis of existing studies for a more comprehensive comparative analysis.

**Table 1:** Summary analysis of existing studies.

**Sr. No**.	**Ref**	**Year**	**Model**	**Features**	**Dataset**	**Results (%)**
1	Bokolo and Liu (2024)	2020	LSTM	HRV from wearables	Wearable devices, HRV	83
2	Zogan et al. (2022)	2020	CNN	Textual features	Twitter, Reddit	93
3	Kim et al. (2020)	2020	Context-DNN	Count vectorization	Patients' data	81
4	Coutts et al. (2020)	2021	LSTM	Textual features	Twitter	74
5	Imel et al. (2024)	2021	BERT-based model	Textual features	Twitter	86
6	Benítez-Andrades et al. (2022)	2021	Psych BERT	Word embedding's	social media text	65
7	Boer et al. (2021)	2022	Bi-LSTM, BERT	TF-IDF and PoS	social media	89
8	Ankalu Vuyyuru et al. (2023)	2022	MLM, BiLSTM	Word embedding's	social media	85
9	Zhu (2023)	2023	BERT	Textual data	Twitter, social media	92
10	Kodati and Tene (2023)	2023	CNN	CBT feature	clinical text data	91
11	Chiong et al. (2021)	2023	MentalBERT	Social media interactions	Facebook, Twitter	76
12	Verma et al. (2023)	2024	DeBERTa,	behavioral features	Sentiment140	89
13	Vajre et al. (2021)	2024	RoBERTa, BERT	Word embedding's	textual data	79

### 2.1 Existing studies of deep learning

Systematic sentiment analysis in mental health datasets is crucial for researchers investigating depression and anxiety, utilizing data sourced from both wearable devices and social media platforms. While some studies have employed transformer models for depression detection, their findings remain limited due to the lack of comprehensive evaluations across diverse datasets, which reduces the generalizability of the results. Furthermore, insufficient reporting on false positive and false negative detection methods weakens the robustness of their conclusions (Verma et al., 2023). Comparative analyses of depression and suicide detection using machine learning and transformer models have also overlooked the effects of dataset bias and class imbalance on model performance. The absence of extensive testing across multiple social media platforms restricts the practical applicability of these approaches, highlighting a significant gap in current research (Bokolo and Liu, 2024).

The research evaluation was limited by a small data sample and unreliable data quality, which decreased the universal applicability of the results regarding wearable technology-based HRV prediction of mental health and overall wellness. Such omissions regarding participant characteristics, including BMI, result in weak generalizations of the study outcomes (Coutts et al., 2020). The proposed deep learning method for depression intensity measurement on social media did not address the negative effects of noisy data on model precision. The model displayed limited ability to recognize diverse populations because the dataset was not diverse (Ghosh and Anwar, 2021). Another study suggested using a hybrid deep learning system to detect depression but failed to examine the security issues related to the vulnerability of social media datasets. The methodology did not account for the fact that language usage varies across social media sites, which created limitations for the model's practical application (Zogan et al., 2022). A deep learning system to identify mental illness from social media did not account for the broad variability of mental symptoms that influence its precision level. The model considered only text information and did not extract corresponding context information regarding user interaction or media post content (Kim et al., 2020). For predicting the risk of depression, deep models were used; however, they failed to consider that multivariable regression could miss identifying non-linear associations between variables in the model. The performance of this model could be degraded by the lack of diversity in real-world data (Baek and Chung, 2020). The study on machine learning and deep learning diagnosis techniques did not include data bias analysis, which could affect the fairness of the model. The lack of clarity in the model process negatively impacted transparency, a crucial aspect of designing mental health applications (Kasanneni et al., 2025).

Important ethical considerations arise when using personal data in a study on predicting mental health consultations from social media posts. The linguistics-based model may have limitations in precision when handling various populations digitally across different platforms (Saha et al., 2022). The final part of the work involved sentiment analysis through the integration of the Bi-LSTM and BERT models in depression prediction. However, it did not consider textual elements such as sarcasm and irony. Furthermore, the model lacks the capacity to perceive non-verbal cues and multimedia elements, as it relies solely on textual information (Boer et al., 2021).

### 2.2 Existing studies of transformer-based models

Transformers possess exceptional power and capability in capturing long-range dependencies and contextual relationships within sequential data through their self-attention mechanisms. This makes them particularly effective for sentiment analysis tasks, where understanding complex language patterns and context is crucial for accurately detecting sentiment polarity and intensity. In literature, the research model faced trust issues because it solely used texts without integrating multiple types of evidence, and the authors withdrew their work (Zeberga et al., 2022). Another study employed transformer-based machine learning for counseling conversation analysis, although it failed to address ethical issues related to the use of therapy data. Prediction accuracy could be improved by incorporating non-verbal cues, as text-based analysis often provides insufficient information (Imel et al., 2024). The assessment of eating disorder-related tweets using machine learning methods alongside BERT models yielded inadequate results because the system design overlooked the diverse expression methods within the text data. Text-only data lacked contextual information about user engagement and multimedia content, which could be vital for understanding the problem (Benítez-Andrades et al., 2022). A transformer-CNN hybrid model for cognitive behavioral therapy assessment in psychological testing did not address the performance inefficiency and resource requirements associated with combining multiple models. The evaluation failed to explore unstructured behavioral and contextual cues, which could enhance both diagnosis accuracy and treatment efficiency (Ankalu Vuyyuru et al., 2023). The analysis of machine learning algorithms alongside deep learning for mental health diagnosis from social media platforms failed to resolve data quality and imbalance issues. Using textual data alone prevented healthcare professionals from accessing multimodal features, which could enhance diagnostic efficiency (Chiong et al., 2021). The social media behavioral analysis system, PsychBERT, did not address potential ethical problems that could arise from using individual data. The text-based analysis approach had a potential drawback because it could not detect important contextual signals that multimedia elements and user activity would provide (Vajre et al., 2021).

A transformer-based deep learning model examined suicidal emotions on social media yet failed to address subtle or hidden expressions because it reduced the system's accuracy level. The approach used only text as its basis while ignoring essential visual and user-related information cues (Kodati and Tene, 2023). The combination of explainable AI with machine learning analyzed Reddit wellbeing but failed to consider linguistic differences and posting contexts, making it difficult to achieve accurate results. The sole reliance on text data prevented the system from discovering vital behavioral and multimodal information that could strengthen prediction accuracy (Thushari et al., 2023). The implementation of RoBERTa-Large and BERT in mental healthcare applications restricted their capacity to handle specialized vocabulary within this domain, thus affecting the system's accuracy. Applications could achieve better performance by incorporating non-verbal cues through text analysis alone (Wu et al., 2024). XAI transformer-based interpretation methods were developed to understand depressed and suicidal user tendencies, although researchers failed to address the difficult nature of detecting subtle expressions. The analysis of text alongside structured data failed to capture non-verbal signals together with situational context, which reduced the accuracy of the interpretation (Malhotra and Jindal, 2024).

## 3 Proposed methodology

**Figure 3:**
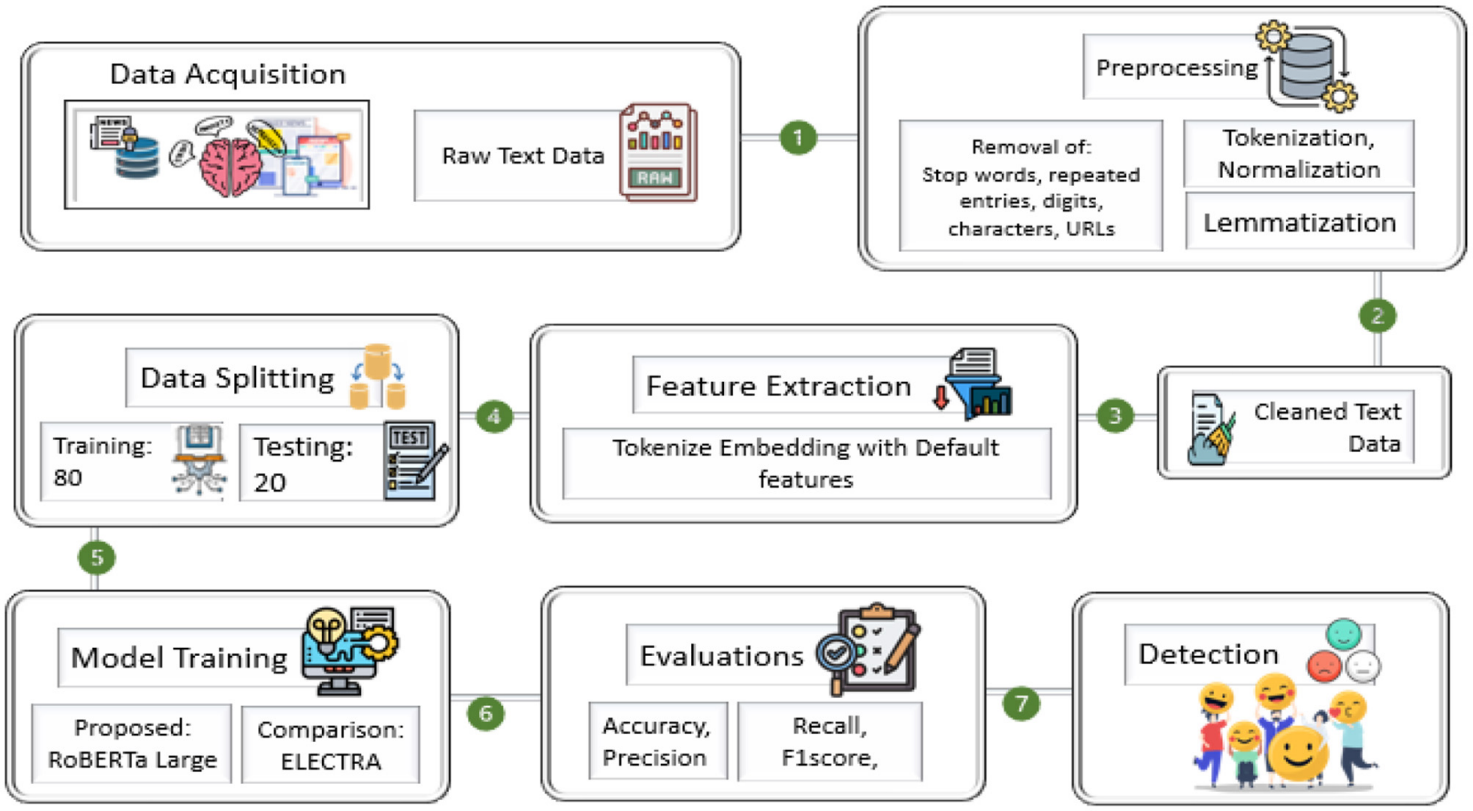
Framework of proposed research methodology.

The steps of the research methodology are shown in Figure 3. First, data collection, preprocessing, feature extraction, and model training are performed using state-of-the-art NLP models. The main steps in data preprocessing are text normalization, removal of stop words, lemmatization, and tokenization, which refine the raw textual data. Using transformer-based architectures such as RoBERTa-Large, sentiment classification is performed accurately, capturing signs of emotions and mental health patterns from user-generated content. The proposed framework aims to enhance sentiment detection accuracy by designing a model that leverages contextual embedding's and deep learning-based classification.

### 3.1 Data preprocessing

In this study, data preprocessing is a crucial step to ensure the accuracy and reliability of sentiment analysis on mental health-related content. The raw text data collected from social media platforms contains a lot of noise, such as irrelevant words, special characters, numbers, and stop words, which can negatively impact the performance of Natural Language Processing (NLP) models (Wu et al., 2023).

Several preprocessing techniques are applied to the textual data to improve its quality (Ahmed et al., 2023). Initially, stop words *S ϵ* {*s*_1_, *s*_2_, ...., *s*_3_, words *w*_*j*_ such as “the” and “is,” which do not carry any useful information in sentiment classification, are removed using function *f*_*stop*_ from dataset *D*, as defined in Equation 1. Table 2 provides a description of the symbols used in the equations for a deeper understanding.


(1) $$\begin{array}{l} d_{i}^{\prime }=\left\{w_{j}|w_{j}\in d_{i},w_{j}\notin S\right\} \end{array}$$

Moreover, set *C* containing digits and characters *c*_*k*_ ϵ *C*, such as punctuation marks or emojis, are also eliminated using function *f*_*char*_ using Equation 2, for textual uniformity and minimum variations in the dataset.


(2) $$\begin{array}{l} d_{i}^{\prime \prime }=\left\{w_{j}|w_{j}\in d_{i}^{\prime },w_{j}\notin C\right\} \end{array}$$

After removing the components that might not be relevant, normalized texts are generated to make the content consistent and minimize the differences among word forms, using function *f*_*norm*_, using Equation 3. This happens by converting all text to lowercase, so that when singling out noise words, they are standardized and independent of case sensitivity.


(3) $$\begin{array}{l} f_{norm}\left(w_{j}\right)=\text{lowercase}\left(w_{j}\right),\;\forall w_{j}\in d_{i}^{\prime \prime } \end{array}$$

After that, we apply lemmatization using *f*_*lemma*_ to lower all words to their base forms to thereby increasing the model's efficiency by limiting redundant word variations. For example, words, such as “running,” “ran” are changed to “run,” where similar semantically words are treated equally, as in Equation 4. Lemmatization preserves the contextual meaning of words while reducing data dimensionality, which aids in better model generalization.


(4) $$\begin{array}{l} d_{i}^{\prime \prime \prime }=\left\{w_{j}^{*}|w_{j}^{*}=f_{lemma}\left(w_{j}\right),w_{j}\in d_{i}^{\prime \prime }\right\} \end{array}$$

Another important step is that the NLP model must initially process the text coverage, which involves tokenization *f*_*token*_, in which the text is previously divided into several words or subwords, allowing the NLP model to understand linguistic patterns, as defined in Equation 5. The model can capture syntactic and semantic relationships by breaking down sentences into meaningful terms.


(5) $$\begin{array}{l} T_{i}=f_{token}\left(d_{i}^{\prime \prime \prime }\right)=\left\{t_{1},t_{2},\ldots ,t_{p}\right\} \end{array}$$

Deep learning architectures, such as transformer-based models, are then used to process the tokens generated after preprocessing. Sentiment analysis becomes more efficient when the data are preprocessed, with the noise first removed, text structures standardized to facilitate further data processing, and only the most valuable features selected for classification. This structured approach enhances model performance in detecting sentiment patterns related to mental health concerns such as anxiety, depression, or emotional distress.

**Table 2:** Explanation of symbols used in equations.

**Symbol**	**Description**	**Symbol**	**Description**
${\text{d}}_{\text{i}}^{\prime }$	Redefined document	*V*	Vocabulary size
**T_i_, P_i_, S_i_ ∈ ℝ^d^**	Token, position, segment embedding of token *x*_*I*_	γ	Residual connection
$\alpha_{\text{T}},\;\alpha_{\text{P}},\;\alpha_{\text{S}}\;\epsilon \;{\mathbb{R}}^{\text{d}}$	Learnable scaling factors are applied to the token, position, and segment embeddings, respectively,	M	Masked token
${\text{W}}_{at}\;\epsilon \;{\mathbb{R}}^{{\text{d}}^{*}\text{d}}$	The weight matrix governing the attention mechanism applied to the previous hidden state $h_{i}^{prev}$	*x*_*t*_, *P*_*t*_ *and s*_*t*_	Token, Position, and Segment embeddings
${\text{b}}_{at}\epsilon \;{\mathbb{R}}^{\text{d}}$	Bias term for the attention mechanism	*M*	Mask for autoregressive tasks
**σ**	Sigmoid activation function ensuring bounded attention weights.	*Q, K, V*	Linear transformations of the input
**Q**_**h**_, **K**_**h**_, **V**_**h**_, ${\text{R}}_{\text{h}}\;\epsilon \;{\mathbb{R}}^{{\text{n}}^{*}{\text{d}}_{\text{k}}}$	Query, key, value, and relative positional embeddings matrix for the *h*−*th* attention head, respectively.	*h*_*t*_	Hidden state at position *t*.
**C**_**h**_	A correction vector is added to the value matrix to refine the output representation	λ	Balances the two losses.
**B**_**h**_	Learned bias matrix applied to the attention logits	D	Data distribution
${\text{h}}_{\text{i}}\epsilon \;{\mathbb{R}}^{\text{d}}$	Input hidden state vector for token *x*_*i*_	$W_{1}\epsilon \;{\mathbb{R}}^{d^{*}d_{ff}};$ $W_{2}\epsilon \;{\mathbb{R}}^{d_{ff}^{*}d};$ $W_{3}\epsilon \;{\mathbb{R}}^{d^{*}d}$	Weight matrices for the feed-forward layers with an additional weight matrix quadratic term,
**d**_**k**_ **and** **d**_**v**_	Dimensionality of the key and value vectors		

### 3.2 Proposed model RoBERTa-Large

**Figure 4:**
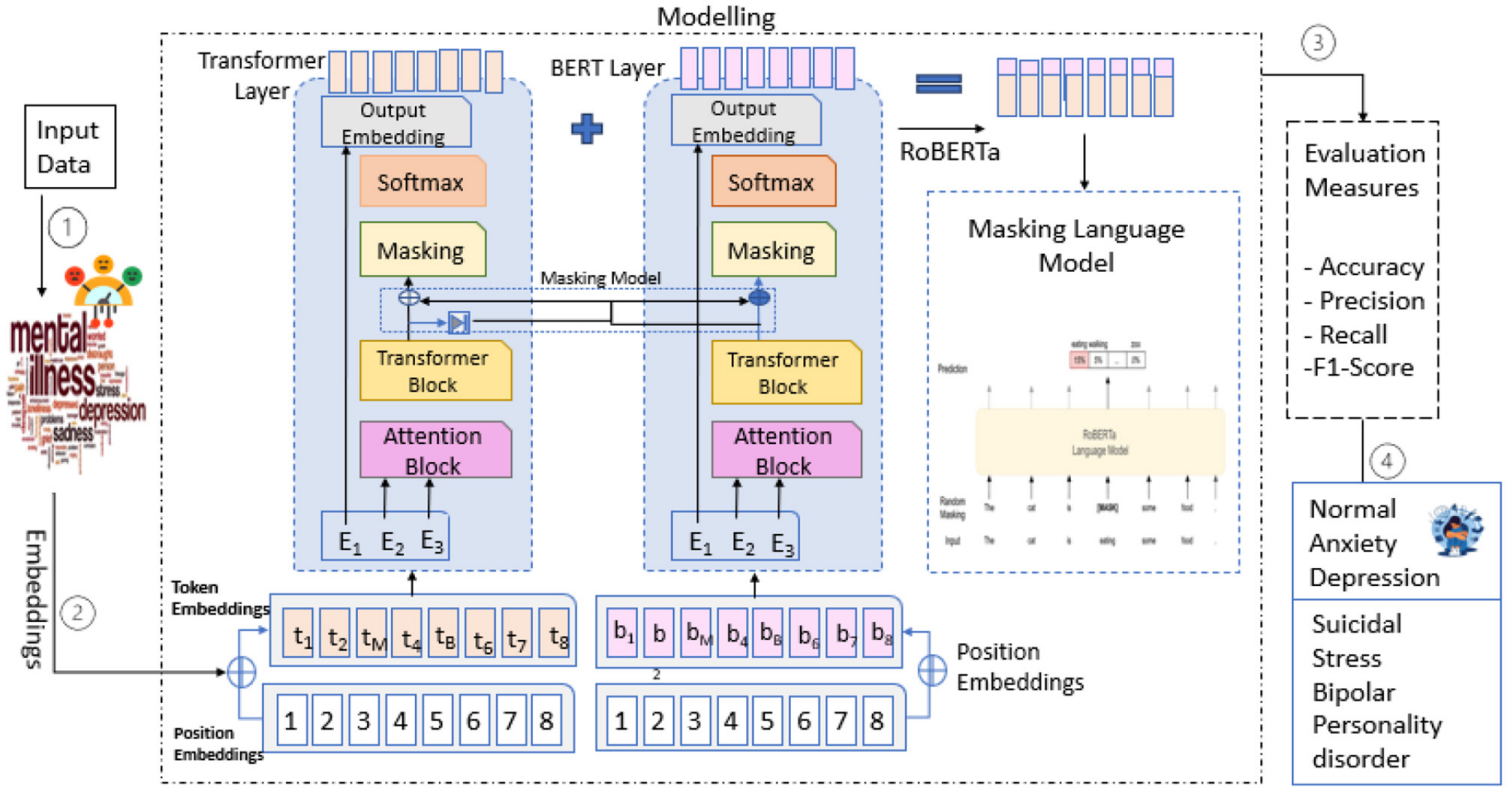
Proposed model architecture.

RoBERTa-Large (Robustly Optimized BERT Pretraining Approach) is an improved transformer-based model that enhances the performance of BERT by refining its pretraining strategy. RoBERTa-Large is based on the BERT architecture but does not include the Next Sentence Prediction (NSP) objective. It utilizes larger batch sizes and is trained on more data with dynamic masking, developed by Facebook AI. RoBERTa-Large's performance is rich in a variety of tasks centered on NLP, ranging from sentiment analysis to classification to mental health detection, as it has 24 transformer layers, 16 attention heads, and 355 million parameters (Youngmin et al., 2024). Due to its ability to leverage fine-grained language representations, it is ideal for analyzing complex textual data (e.g., user-generated content about mental health discussions), as proposed by the model architecture defined in Figure 4.

#### 3.2.1 Input embedding's layers

In RoBERTa-Large, the input embedding is responsible for converting input tokens into high-dimensional, dense vectors. These vectors are formed by combining three components.

*Token embeddings:* Each token in the input is mapped to a fixed-size vector, representing a learned embedding of the token in the embedding space, as defined in Equation 6.

*Position embeddings:* Since the transformer core design does not capture the position of tokens within the sequence, position embeddings must be added to facilitate the understanding of the token positions within the sequence.

*Segment embeddings:* If the task involves a sentence pair (e.g., question answering), segment embeddings help identify the two sentences in the pair separately. These embeddings are then combined and fed into the transformer layers. This transformation enables the model to learn about information such as semantics (the meaning of tokens) as well as syntax (the arrangement of tokens in a specific order).


(6) $$\begin{array}{l} \left[E_{i}=\left(\alpha_{T}\cdot T_{i}+\alpha_{P}\cdot P_{i}+\alpha_{S}\cdot S_{i}\right)\cdot \sigma W_{at}\cdot h_{i}^{prev}+b_{at}\right] \end{array}$$

#### 3.2.2 Multi-head self-attention

RoBERTa-Large, which uses a self-attention mechanism for input embedding's that are projected to several attention heads. There is a different aspect of the relationship between tokens that each head learns. Specifically, it learns a query, a key, and a value for each token. The query of a token is compared to the keys of all other tokens, and attention is computed for each token in the sequence (Chen et al., 2024). A multi-head attention mechanism allows the model to consider relationships and contextual details in parallel, but not at such a fine-grained level as to gain significant expression. The attention computation results of each head are concatenated and then passed through a final linear layer to obtain the attention output calculated using Equation 7.


(7) $$\begin{array}{l} A_{h}=\text{softmax}\left(\frac{\left(Q_{h}+R_{h}\right){\left(K_{h}+R_{h}\right)}^{⊤}+B_{h}}{\sqrt{d_{k}}}\right)\cdot \left(V_{h}+C_{h}\right) \end{array}$$

#### 3.2.3 Feed forwarded network

The combination of the attention layer and the FFN processes the result from the layer. The FFN has two linear layers and one ReLU in between. This part of the network can be a non-linear transformation, which allows the model to better learn more intricate patterns in the data, as shown in Equation 8. Each token's representation is hence transformed independently by the FFN. This is very important because, by examining the information present in the self-attention layers, the FFN enables RoBERTa-Large to establish more powerful, higher-level non-linear relationships.


(8) $$\begin{array}{r} h_{i}^{FFN}=\text{ReLU}\left(\left(h_{i}\cdot W_{1}+b_{1}\right)\cdot \left(h_{i}\cdot W_{3}+b_{3}\right)\right)\cdot W_{2}+b_{2}+\gamma \cdot \\ \left(h_{i}-h_{i}^{prev}\right) \end{array}$$

#### 3.2.4 Residual connection and layer normalization

After each sublayer (attention layer or feed-forward network), RoBERTa-Large makes use of residual connections to facilitate gradient flow during training. Through these connections, the input can be added to the output of the sublayer without passing through the sub-layers. It prevents the network from vanishing gradients, allowing the training of deeper models. Layer normalization is executed after adding the residual connection. This allows training to be stabilized by normalizing the hidden states over the layer's output, as in Equation 9.


(9) $$\begin{array}{r} h_{i}^{nr}=\text{LayerNorm}\left(h_{i}+W_{r}\cdot \text{ReLU}\left(h_{i}\cdot W_{1}+b_{1}\right)+b_{r}\right)\cdot \\ \sigma \left(W_{2}\cdot h_{i}+b_{2}\right) \end{array}$$

#### 3.2.5 Masked language modeling (MLM) objective

In pretraining, some of the input tokens are randomly masked, computed using Equation 10. The task in the model is to predict the masked tokens based on the context provided by the surrounding tokens, thereby learning word relationships within a sentence and their context. A standard cross-entropy loss is minimized between the predicted masked tokens and the actual tokens. However, this helps the model learn a robust representation of language, allowing it to generalize very well to downstream NLP tasks as well.


(10) $$\begin{array}{l} L_{MLM}\;=\;-\underset{i\epsilon M}{\prod }log\left(\frac{exp\left(W_{out}.h_{i}^{LM}+b_{out}\right)}{\prod_{j=1}^{V}exp\left(W_{out}.h_{i}^{LM}+b_{out}\right)}\right) \end{array}$$

### 3.3 Proposed model ELECTRA

**Figure 5:**
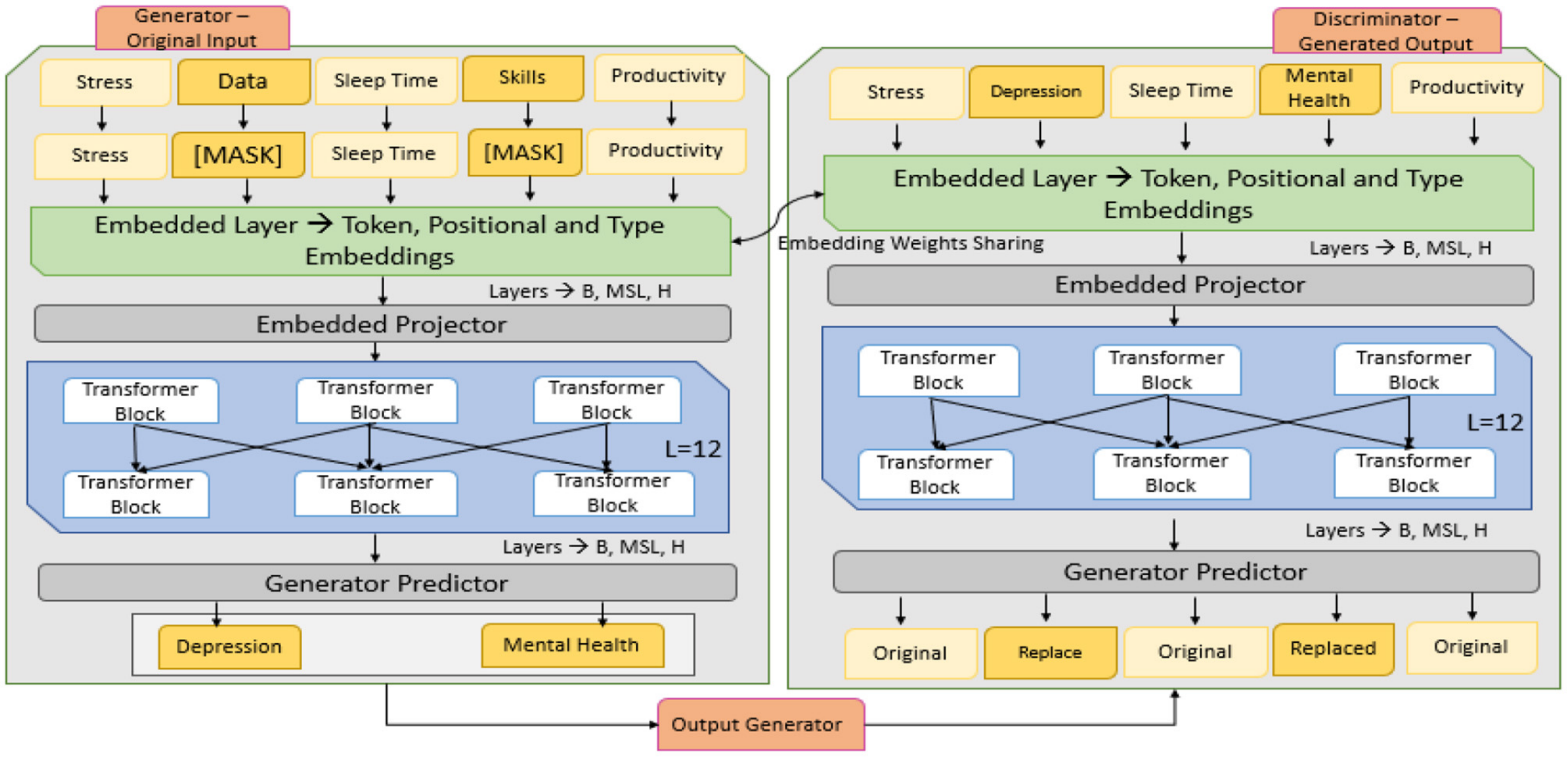
Architecture of ELECTRA model.

Efficiently learning an Encoder that Classifies Token Replacements Accurately (ELECTRA) uses a pre-training approach called Replaced Token Detection (RTD). ELECTRA does not predict masked tokens; instead, it learns to distinguish masked tokens from plausible replacements that it generates using a small generator network, as shown in the working architecture defined in Figure 5. The ELECTRA model consists of two components: Generator *G* and Discriminator *D*. The generator is a small, masked language model (MLM) model that generates the masked tokens of a given input sequence. It produces mismatched versions of input but generates plausible replacements for masked tokens. The generator is trained to replace the tokens with replacements that would be generated by the discriminator, a larger transformer. ELECTRA is more efficient and effective than BERT, as it can evaluate each token in the sequence, not just the masked ones (Ahmed et al., 2024). Both the generator and the discriminator are trained jointly, but only the discriminator is utilized in downstream tasks, such as sentiment analysis.

#### 3.3.1 Input embedding layer

The tokens they use can be words, subwords, or any other type of token, and they convert these into continuous vector representations, such as those obtained using pre-trained word embedding's, as shown in Equation 11. Additionally, it modifies the input by appending positional encodings to account for the order of the input sequence.


(11) $$\begin{array}{l} E=E_{token}\left(x_{t}\right)+{\text{E}}_{position}\left(t\right)+{\text{E}}_{segment}\left(s_{t}\right) \end{array}$$

#### 3.3.2 Transformer encoder layers

This consists of a stack of transformer encoder blocks that take an embedded input. The layers in these models utilize self-attention and feedforward networks on top of the sequence, handling multi-head self-attention to capture dependencies between tokens within the sequence, as computed in Equation 12. Based on the refined representation of the input, the encoder outputs the text inputs.


(12) $$\begin{array}{l} \text{Attention}\left(Q,K,V\right)=\text{softmax}\left(\frac{QK^{T}}{\sqrt{d_{k}}}+M\right)V \end{array}$$

#### 3.3.3 Generator output layer

In ELECTRA, the generator is an auxiliary MLM that predicts missing text using Equation 13. It learns to produce plausible candidate tokens for the masked positions in the input sequence, matching the existing tokens.


(13) $$\begin{array}{l} P_{G}\left(x_{t}|\tilde{x}\right)=\text{softmax}\left(W_{G}\cdot \text{LayerNorm}\left(h_{t}\right)+b_{G}\right) \end{array}$$

#### 3.3.4 Discriminator output layer

The discriminator is responsible for identifying real tokens (those originating from the original data) and fake tokens (those generated by the generator), as defined in Equation 14. It provides a probabilistic answer for each token, indicating whether it is real or fake.


(14) $$\begin{array}{l} D\left({\hat{x}}_{t}|x\right)=\sigma \left(W_{D}\cdot \text{GELU}\left(W_{po}\cdot h_{t}+b_{po}\right)+b_{D}\right) \end{array}$$

#### 3.3.5 Joint training objective

The two tasks are trained jointly: one for the generator (the MLM task, predicting masked tokens) and the other for the discriminator (classifying tokens as real or fake using RTD losses), as shown in Equation 15. The goal is to maximize the discriminator's input to identify fake tokens while minimizing the error made by the generator in producing realistic tokens.


(15) $$\begin{array}{r} L_{ELECTRA}=E_{x\sim 𝔻}[\sum_{t\in M}-\log P_{G}\left(x_{t}|\tilde{x}\right)+\lambda \sum_{t=1}^{T}-I\left(x_{t}={\hat{x}}_{t}\right)\\ \log D\left({\hat{x}}_{t}|x\right)-I\left(x_{t}\neq {\hat{x}}_{t}\right)\log\left(1-D\left({\hat{x}}_{t}|x\right)\right)] \end{array}$$

### 3.4 Dataset

For the experiments, the dataset is sourced from the Kaggle website, which contains a collection of mental health-related statements from various datasets, including the 3k Conversations Dataset for Chatbot, Depression Reddit Cleaned, Human Stress Prediction, and others. The dataset content is based on reviews generated by users of online platforms such as Reddit and Twitter but is annotated with one of the seven mental health statuses, including normal, depression, suicidal ideation, anxiety, stress, bipolar, and personality disorder. Each entry consists of a unique identifier, a text statement, and a corresponding mental health label. This dataset provides a substantial amount of data for machine learning models to utilize for sentiment analysis and chatbot development, which can aid in early detection and support for mental health conditions.

### 3.5 Baseline models

For the comparative analysis, several state-of-the-art deep learning models are considered for detecting complex patterns.

#### 3.5.1 LSTM

The LSTM architecture is simplified by the GRU, which uses fewer gates, making it faster to compute and easier to optimize while still addressing vanishing gradient problems. The LSTM has an input, output, and forget gate that regulate the flow of information in a robust manner, allowing for the learning of long-term dependencies.

#### 3.5.2 Bi-LSTM

From the context provided, BiLSTM enhances the data processing capability of LSTM by processing data in both forward and backward directions, providing the network with significantly more information to work with. This is particularly suitable for tasks that are highly dependent on context, whether in the past or the future.

#### 3.5.3 GRU

A Gated Recurrent Unit (GRU) is a variant of the standard LSTM network that has streamlined the mechanism for processing sequential data for NLP tasks. The vanishing gradient problem is addressed by GRUs, which have two key gates: the update gate and the reset gate. These gates enable the model to determine which information should be retained and which should be discarded, thereby allowing it to better capture the dependencies of information across time steps, as observed in text data. That is why GRUs are specifically designed for tasks that rely on understanding context and temporal relationships in text, including language modeling, text generation, and sentiment analysis.

### 3.6 Implementation tools and utilities

The proposed model was developed and fine-tuned using widely adopted deep learning and NLP libraries, including PyTorch and the Hugging Face Transformers framework. Data handling and preprocessing were performed using Pandas and NumPy, while scikit-learn was utilized for evaluation metrics. Visualization and interpretability were supported through Matplotlib and Seaborn, as summarized in Table 3. The training and evaluation were conducted on a GPU-enabled environment using Google Colab to ensure efficient computation.

**Table 3:** Explanation of experimental setup and resources.

**Category**	**Libraries**	**Version**	**Purpose**
Deep learning framework	PyTorch	2.0.1	Core framework for building, training, and deploying the RoBERTa model.
Transformer models	Hugging Face Transformers	4.30.2	Provides pretrained RoBERTa models, tokenizers, and utilities for fine-tuning.
Data handling	Pandas	2.0.3	For loading, cleaning, and managing textual datasets in tabular format.
Numerical computation	NumPy	1.24.3	Efficient numerical operations, array manipulation, and preprocessing support.
Machine learning utilities	Scikit-learn	1.3.0	Train-test split, metrics (accuracy, precision, recall, and F1 score), and confusion matrix.
Visualization	Matplotlib	3.7.2	For plotting training curves, confusion matrices, error analysis, and other visualizations.
	Seaborn	0.12.2	High-level visualization for performance metrics and distributions.
Experiment tracking	TensorBoard	2.13.0	Logging and visualization of training metrics.
	Weights & Biases (wandb)	0.15.4	Tracking training runs, hyperparameters, and performance comparison.
Text preprocessing	NLTK	3.8.1	Tokenization, stopword removal, and lemmatization
	spaCy	3.5.3	Advanced linguistic preprocessing (POS, NER, and dependency parsing).

## 4 Results and discussion

The empirical analysis-based results are discussed in this section. First, we discuss the descriptive perspective of the datasets, sharing exploratory data analysis, and then the predictive results using applied deep learning models are discussed.

### 4.1 Descriptive analysis

The dataset is compiled from comments shared by students, focusing on their mental health experiences and the impact of education and physical activities on their wellbeing. Students were encouraged to express their thoughts on how various activities impacted their mental state in terms of stress, anxiety, depression, and overall psychological resilience. The responses reflect a wide range of emotions and sentiments related to student activities, academic pressures, and personal challenges. These textual responses are structured and labeled in the dataset, serving as input for sentiment analysis and mental health assessment. Such data can be used by AI models to identify patterns and trends related to the mental wellbeing of students.

**Figure 6:**
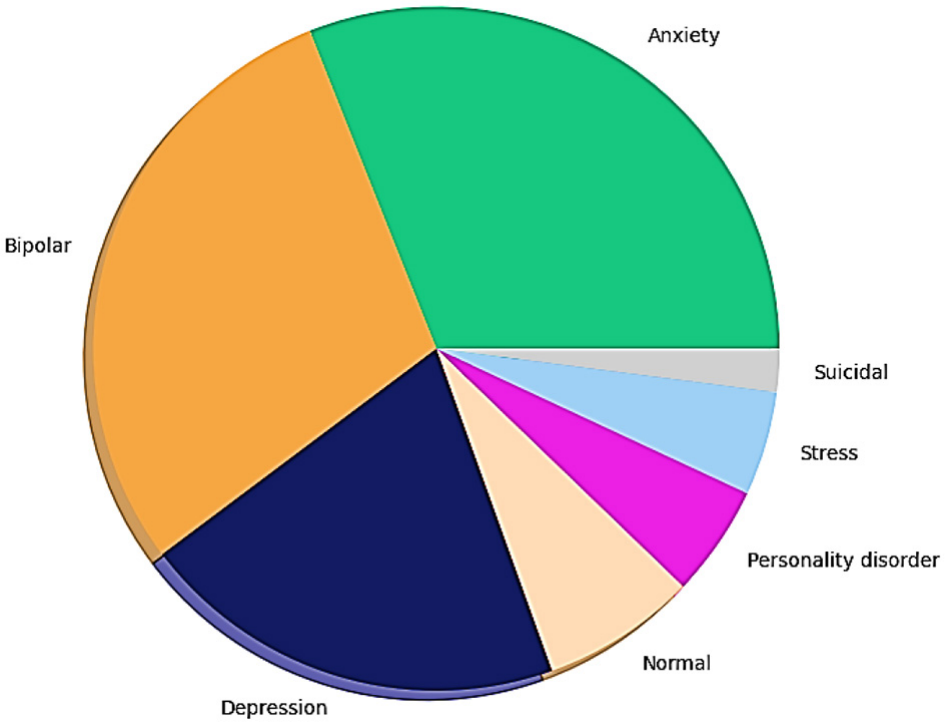
Count of mental health status.

The pie chart in Figure 6 provides crucial insights into the dataset's composition regarding various mental health statuses. The dataset comprised seven classes with the following distribution: normal (16,351 samples), depression (15,404 samples), suicidal ideation (10,653 samples), anxiety (3,888 samples), bipolar disorder (2,877 samples), stress (2,669 samples), and personality disorder (1,201 samples). The most frequently occurring labels are “depression and suicidal tendencies,” suggesting that the collected data are more likely to involve these labels.

Significantly also represented is anxiety about its widespread presence in mental health discussions. On the other hand, possible class imbalance is higher in categories like bipolar disorder and personality disorder, and such classes are rare, whereas the described category is common. The dataset distribution also matches with that of real-life trends, where depression and anxiety are more prevalent on social media, and the prevalence of disorders such as bipolar and personality disorders might be less represented, potentially because of stigma and less self-reporting on public forums. For robust NLP models for sentiment analysis, the distribution of labels must be known. It aids in determining the need for techniques to balance feature engineering methods and evaluation metrics to achieve favorable model performance across all categories of mental health. The skewed distribution also suggests that the model should be evaluated carefully, considering a precision–recall tradeoff to ensure that predictions for the minority class are reliable.

**Figure 7:**
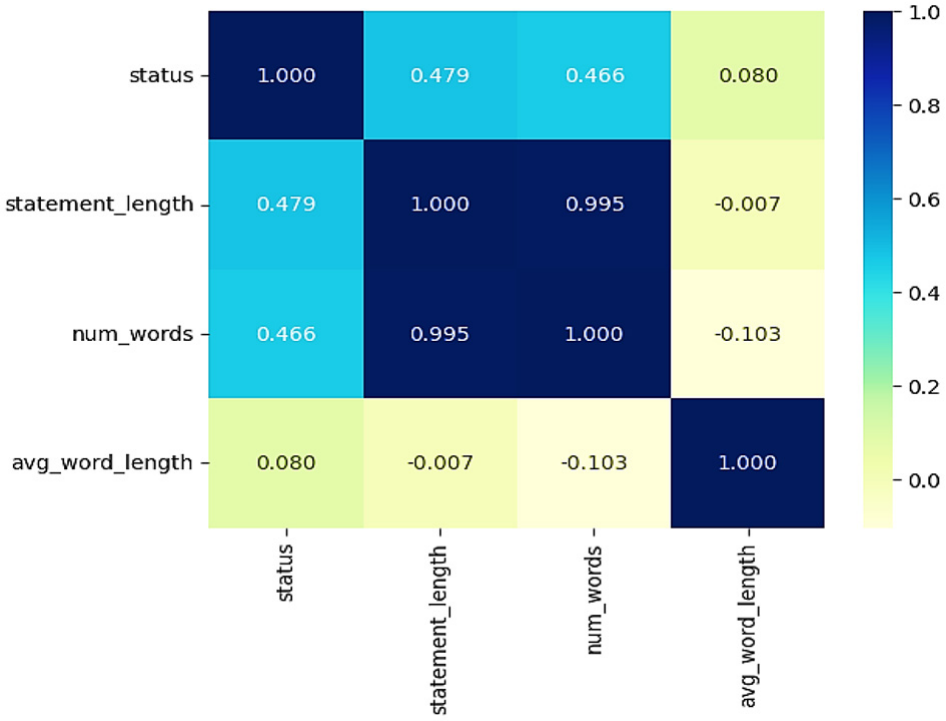
Correlation heatmap information about the relationship between the various textual features.

The correlation heatmap in Figure 7 provides insights into the relationships between the various textual features in the dataset. Statement length (*r* = 0.479) and number of words (*r* = 0.466) exhibit a moderate positive correlation with the status variable, indicating that different mental health conditions may require longer statements. Moreover, we observe that the feature statement length and the number of words clearly correlate (*r* = 0.995), as longer statements obviously contain more words. Average word length, however, has a very weak correlation with all other features, suggesting that there is little difference in word complexity within mental health conditions. This proves that textual attributes can be utilized for sentiment analysis in mental health classification.

**Figure 8:**
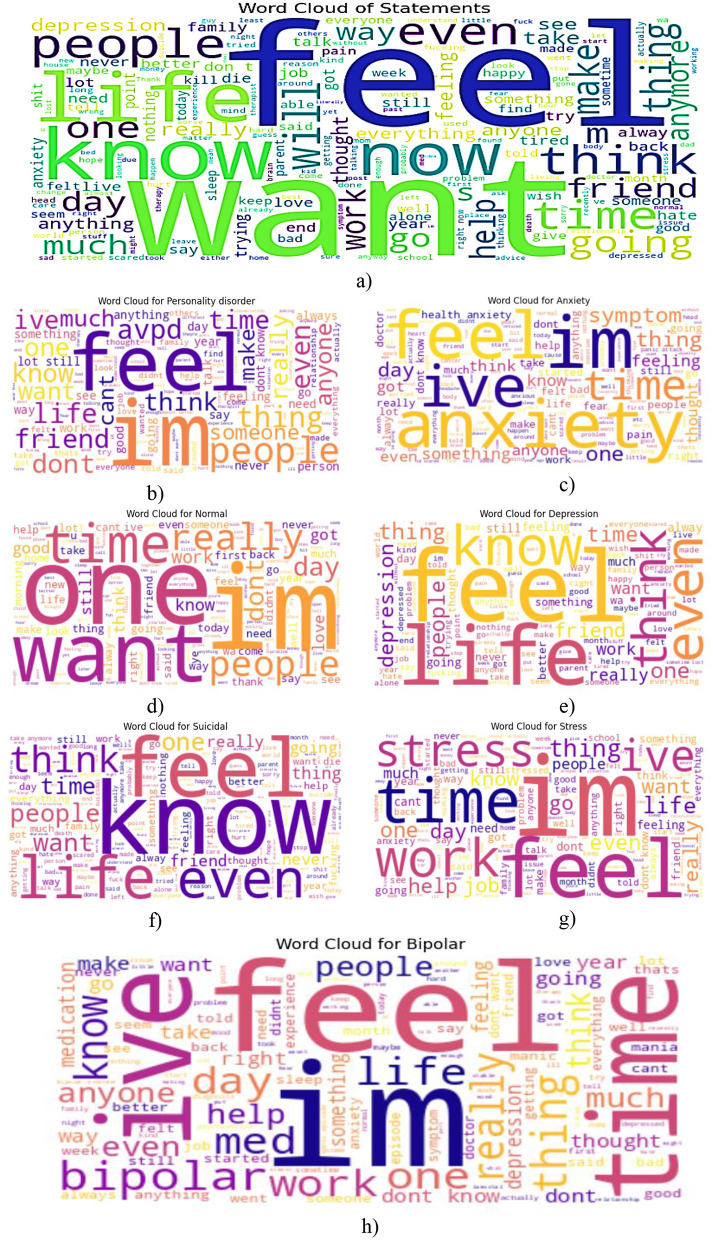
Word cloud of most frequent words from each class label. **(a)** Overall, **(b)** personality disorder, **(c)** Anxiety, **(d)** normal status category, **(e)** depression, **(f)** suicidal ideation, **(g)** stress-related word cloud, **(h)** bipolar.

The word clouds in Figure 8, from the provided visualizations, indicate the words most frequently used in relation to mental health statuses. The word clouds provide an overall linguistic representation of the user-generated content's depiction of mental health status.

*Overall, in Figure (a)*, they feel, want, know, think, live, and other words that dominate this word cloud represent the entire dataset. These words fit within the sphere of introspective and emotional discussions about the disease. “Help” and “work” are also common words that indicate how external support and occupational stress may play a role in shaping the language of mental health.

*Personality Disorder in Figure (b):* Words such as “feel,” “life,” “help,” and “think” are scattered within the personality disorder category. This means that people in this category tend to consider their emotions, relationships, and the self. Help implies that many call for external aid or help.

*Anxiety in Figure (c):* The word cloud related to anxiety includes “feel,” “think,” “know,” “life,” and “anxiety.” The overthinking that comes through thinking and knowing suggests that anxiety is prominent here. Through the repetition of feeling, the pronounced repetition of feeling highlights how anxious thinking is associated with emotional distress, implying the need for reassurance or professional assistance.

In this *normal status category, as shown in Figure (d)*, the word cloud displays words such as “feel,” “life,” “think,” and “know,” and does not carry the heavy emotional weight of the other categories. This category comprises statements that, in most cases, are not particularly emotional, yet they still involve a neutral or positive discussion. This implies that their terms are balanced and do not have any significant mental health concerns or conversational patterns.

*Depression in Figure (e):* Words such as “feel,” “life,” “want,” “help,” and “think” occur most in the word cloud of the depression-related words. Life and want are two things that indicate existential feelings, such as longing or hopelessness. The prominence placed on help highlights the importance of support systems for individuals who are depressed.

*Suicidal ideation in Figure (f):* Words in the suicidal ideation category are prominent, including “want” and “life,” and “know” and “feel,” indicative of distressing thoughts and existential, and feeling words. “End” is also used in these statements, suggesting that there is emotional chaos of the worst kind imaginable. This is a pattern of behavior that requires mental health interventions urgently, by people saying such things. Included in the *stress-*related word cloud *in Figure (g)* are the words “stress,” “work,” “time,” and “life,” which all relate to it. This implies that work and time, if they are dominant, are primary factors leading to stress. Therefore, in addition to this, thinking and knowing indicate cognitive strain, where people think about and understand their stressful situations.

*Bipolar in Figure (h):* Among the most prominent words in the bipolar disorder category are “go,” “time,” “work,” “feel,” and “help.” Bipolar mood swings are often signaled by fluctuating energy levels and erratic thought processes, both of which point to “go” and “time.”

This analysis, which utilizes word clouds to explore the frequency of the most common words in a corpus, facilitates an understanding of the linguistic markers associated with different mental health conditions.

### 4.2 Proposed model results

The proposed model was applied to the student mental health dataset, yielding reliable detection of key psychological patterns and risk factors. The findings highlight the model's potential in supporting early identification and intervention for students' wellbeing.

#### 4.2.1 LLM model RoBERTa

The RoBERTa-Large model is characterized by a set of carefully tuned hyperparameters that optimize its performance for natural language processing tasks. Table 4 summarizes the key hyperparameters of the RoBERTa-Large model. To reduce misclassification errors between similar classes, such as depression and bipolar, a weighted cross-entropy loss was used with class-specific weights obtained based on their occurrence frequencies. This hyperparameter tuning ensured that boxes belonging to minority classes have a larger penalty during training, which improves the model's sensitivity toward bipolar instances without affecting performance on depression. The hyperparameters were tuned using a grid search strategy to identify the optimal configuration and enhance the model's generalizability.

**Table 4:** Hyperparameter setting of proposed model.

**Parameter**	**Values**	**Description**
Layers	24	The number of transformer encoder layers in the model.
Hidden size	1,024	Dimensionality of the hidden states and embeddings.
Attention heads	16	Number of self-attention heads in each multi-head attention layer.
Feed-forward size	4,096	Dimensionality of the intermediate layer in the position-wise feed-forward network.
Max sequence length	512	The maximum number of tokens the model can process in a single input sequence.
Vocabulary size	265	Size of the token vocabulary used by the model.
Dropout	0.5	The dropout rate is applied to prevent overfitting during training.
Attention dropout	0.5	Dropout rate applied to the attention weights.
Activation function	GELU	Activation function used in the feed-forward network (Gaussian Error Linear Unit).
Learning rate	3e-5	Initial learning rate used during pretraining.
Batch size	8,192	Batch size used during pretraining.
Weight decay	0.001	L2 regularization was applied to the model weights.
Warmup steps	24,000	Number of warm-up steps for learning rate scheduling.
Total steps	~500,000	Total number of training steps during pretraining.
Log function	Weighted cross-entropy	Used to penalize misclassification of minority classes more heavily.
Class weights	Inverse class frequency	Weights are assigned proportionally to the inverse frequency of each class.
Sampler	Weighted random sampler	Ensure balanced mini-batches by oversampling minority classes.
Adam epsilon	1e−9	Term added to the denominator for numerical stability in the Adam optimizer.
Adam beta1	0.57	Exponential decay rate for the first moment estimates in the Adam optimizer.
Adam beta2	0.98	Exponential decay rate for the second moment estimates in the Adam optimizer.
Masking probability	20%	Percentage of tokens masked during the masked language modeling (MLM) objective.
Gradient clipping	1.0	Maximum gradient norm for gradient clipping to prevent exploding gradients.

**Figure 9:**
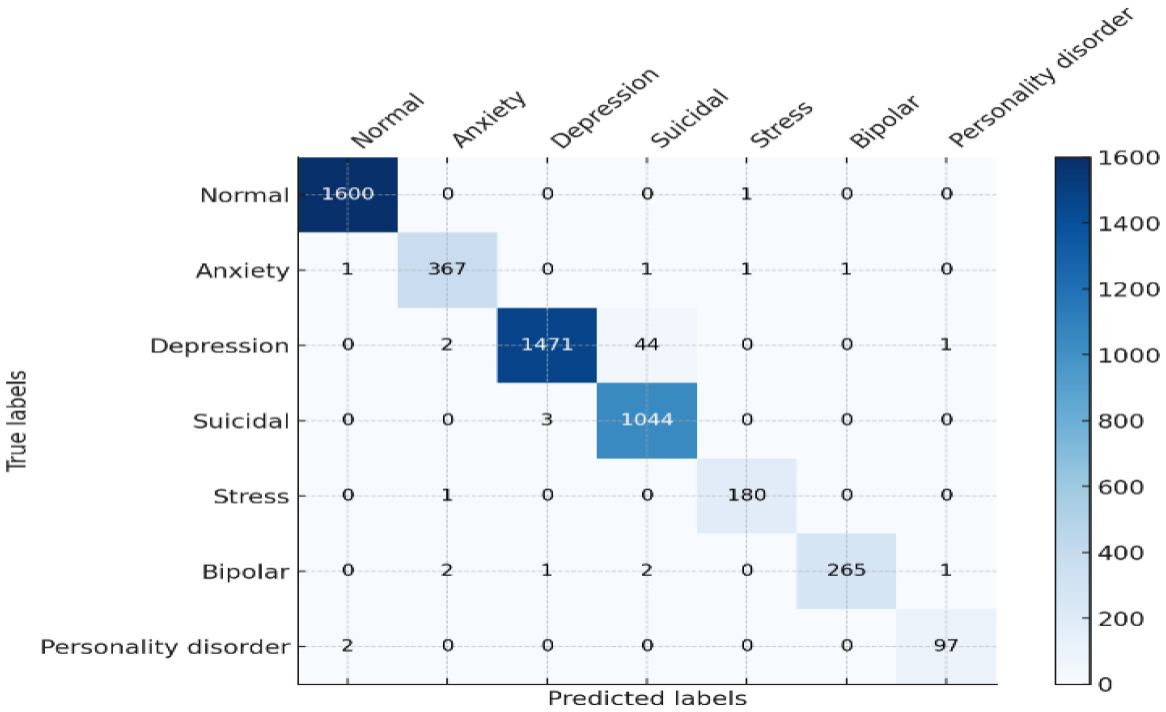
Confusion matrix of proposed model.

Regarding the monitoring and evaluation of mental health in students, leveraging an intensive model (such as RoBERTa-Large-LARGE) in intelligent artificial systems is a new and transformative approach. The deployment of this model in detecting mental conditions using sentiment analysis provides valuable insights into its capabilities and potential applications. The model has an accuracy of 97%, a precision of 95%, a recall of 91%, and an F1 score of 94%. Together, these metrics provide a representation of a superlative model that can reliably and precisely predict the classification of mental health conditions, relying on sentiment data information. This precision is so high as to minimize the risks of false positives, which is especially important when dealing with mental health assessments so that students are not put under unnecessary stress, as shown in the comprehensive analysis of the confusion matrix in Figure 9.

**Figure 10:**
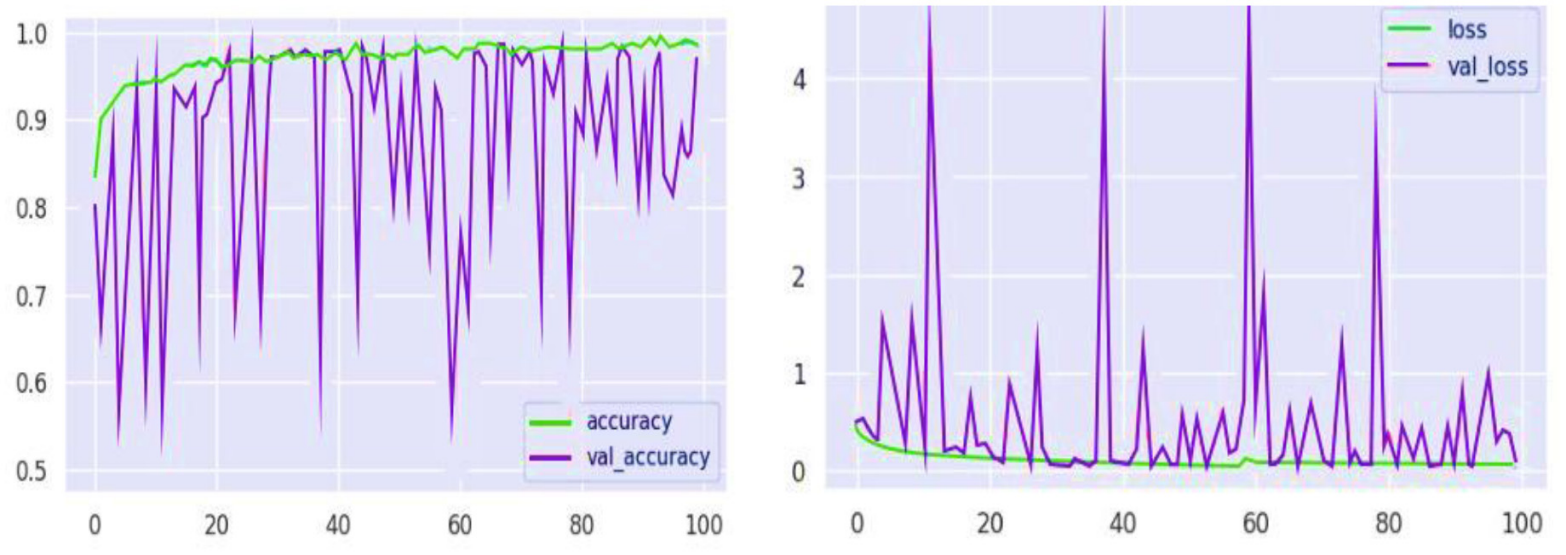
Model performance analysis of loss and accuracy graphs.

Similarly, the high recall rate highlights the fact that models can accurately identify true cases of mental health problems with a low likelihood of false negatives. Analysis of the confusion matrix shows that there are significant true positives in terms of diagnosing conditions such as anxiety, bipolar disorder, and depression, with large numbers on the matrix's diagonal. The analysis of training and validation further represents a path of convergence whereby accuracy reaches the upper thresholds, proving that the model is effective at learning. The loss graph in Figure 10, however, is random mainly in the validation loss, which indicates some drops in the model's performance on the validation data at some point(s). Similarly, these spikes are an important indicator of how responsive the model is to specific features or data anomalies, and they aid in further optimization.

Collectively, the results from model performance demonstrate that these are robust and adaptable AI models. Based on sentiment analysis, the RoBERTa-LARGE model has been shown to accurately and efficiently identify mental health conditions among students, indicating its potential for use in real-world applications. However, the insights suggest that the model must be further refined and recalibrated continuously to achieve higher precision and generalization on other datasets in order to be effective in different environments.

#### 4.2.2 ELECTRA model

**Figure 11:**
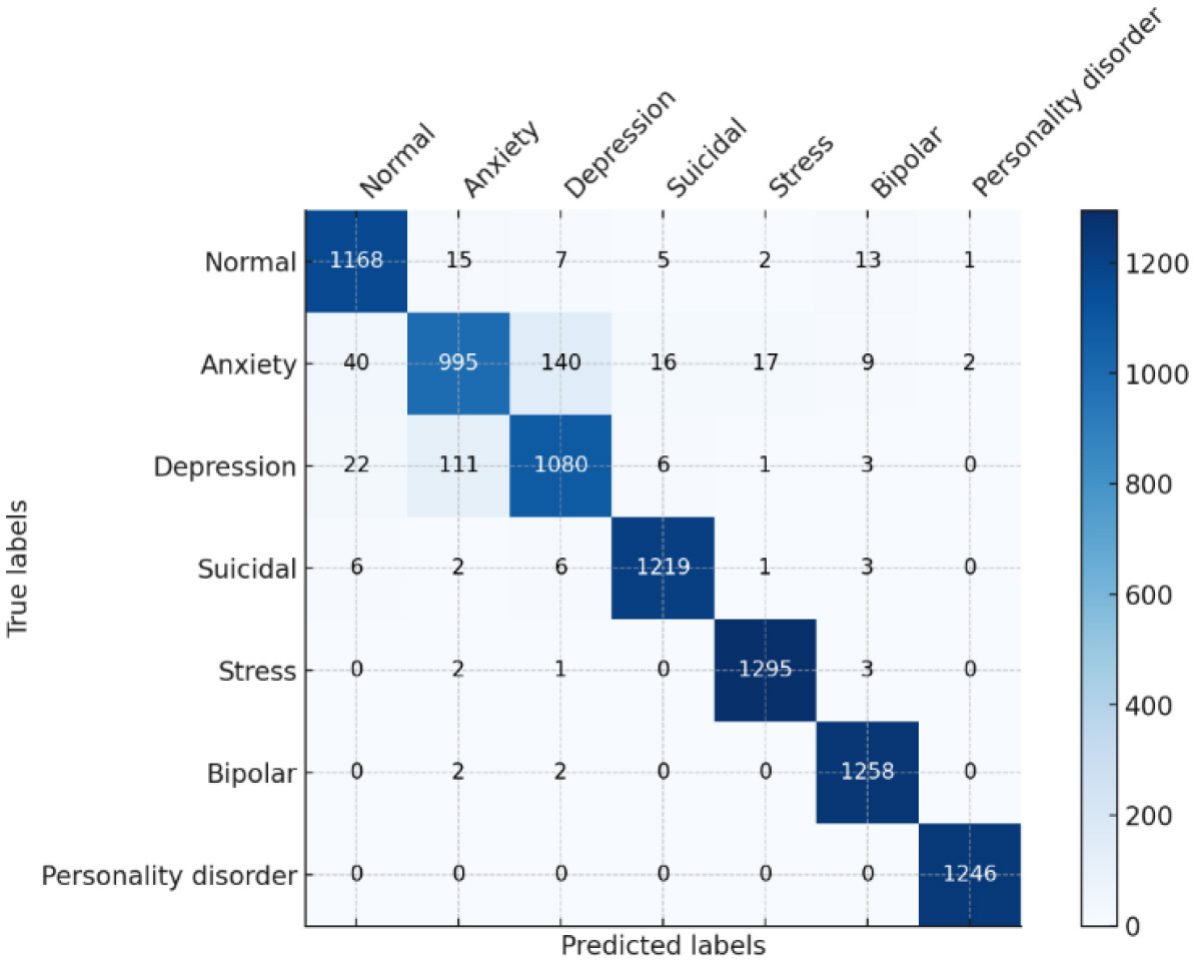
Confusion matrix of ELECTRA model.

The baseline results of the ELECTRA model in predicting the mental health of students via sentiment analysis prove to be quite strong, with all values of overall accuracy, precision, recall, and F1 score equaling 91%. The model's proficiency in accurately classifying the sentiments in categories such as anxiety, bipolar, depression, normal, personality disorder, stress, and suicidal ideation. Regarding all metrics, this high level of performance indicates that the ELECTRA model effectively interprets small-scale language to identify various mental health conditions, making it an appropriate instrument for early detection and monitoring in academic environments. A detailed view of the model's performance for each class is provided in the confusion matrix. For example, numbers such as “normal” and “suicidal,” which are indicated by the large numbers on the diagonal, indicate a high degree of accuracy of the model in predicting these two states with very few misclassifications, as shown in Figure 11. However, there are unresolved questions as well, for example, between “bipolar” and “depression” or “anxiety” and “stress,” that are highly similar in a clinical or linguistic sense, and which increase the misclassification rates. As a result, this implies that the model, in general, is effective, although it may need to be refined or provided with more training data to better differentiate similar conditions.

**Figure 12:**
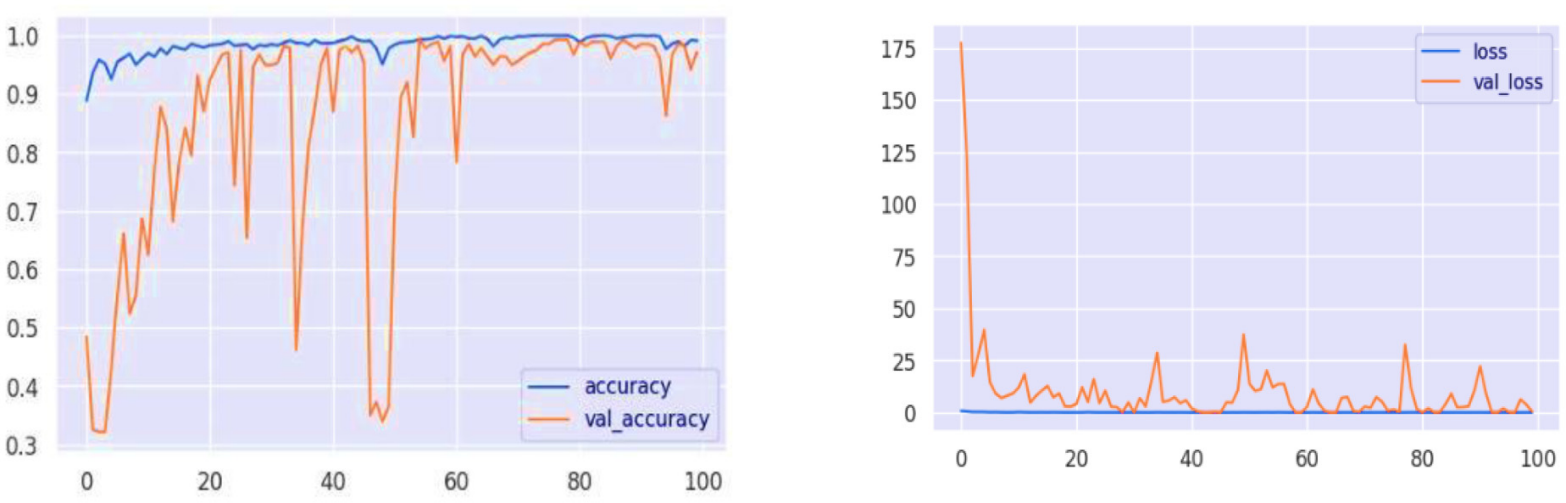
Model performance analysis of loss and accuracy graphs.

The model's learning progress through epochs is visualized by its accuracy and loss graphs, as shown in Figure 12. The accuracy graph remains stable as it converges to high accuracy with the training data, exhibiting minimal overfitting. It is a positive indicator because the validation accuracy closely tracks with the training accuracy, and the model appears to be generalized to new data. The loss graph indicates that the loss is in a downward trend, particularly for the validation loss, with sharp declines following the initial fluctuations. The increase in accuracy confirms this reduction in loss, indicating that the model was reducing error over time. The plot clearly demonstrates the good generalization property that the training and validation performance of the ELECTRA model are well-matched. With that, the model performs well both in learning and operating on new, unseen data it faces—a very desirable feature for practical use in many possibly disparate learning environments. With these small variations in accuracy and loss during validation, there is one area where the model can be improved to make it more robust for real-world data variation through regularization and hyperparameter tuning.

### 4.3 Comparison of the proposed model with the baseline models

By examining confusion matrices that predict the mental health of students using ELECTRA, GRU, LSTM, and Bi-LSTM models, this study provides a clear understanding of the strengths and limitations of these approaches. These models were subsequently evaluated under various conditions, annotated as anxiety, bipolar, depression, normal, personality disorder, stress, and suicidal ideation, which show the predictive capabilities and inadequacies by means of rates of correct and incorrect classification for the model.

#### 4.3.1 GRU model

**Figure 13:**
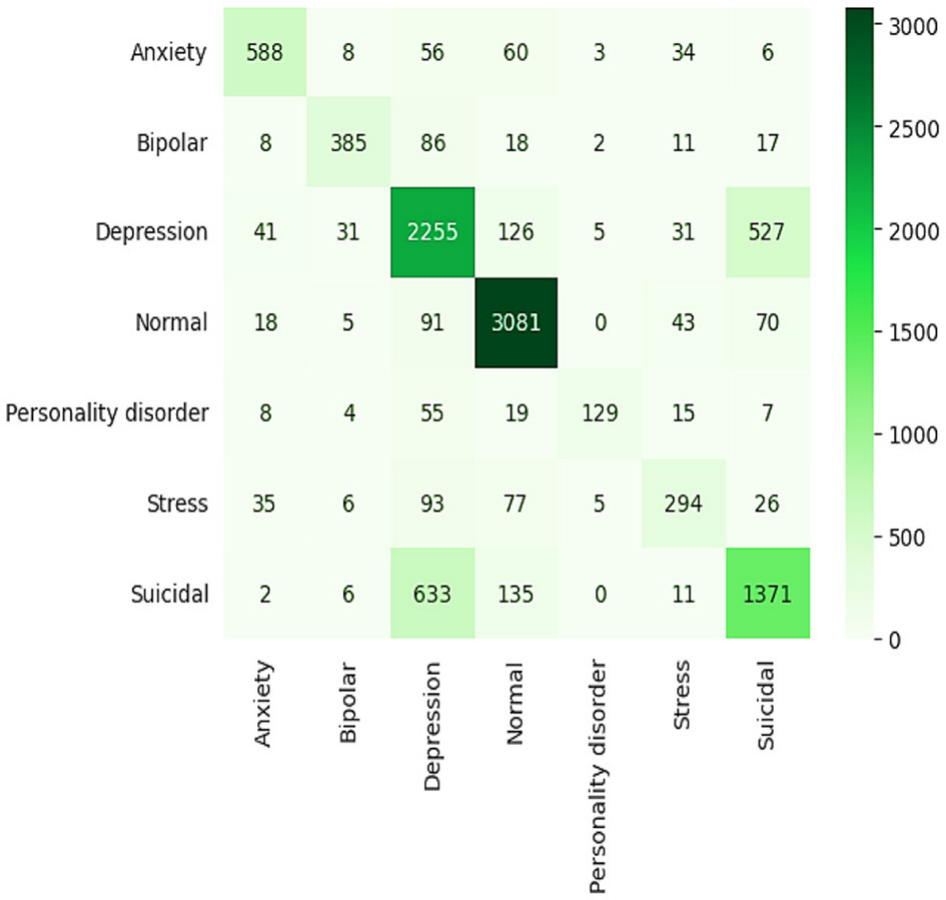
Confusion matrix of GRU model.

Results showing 77% accuracy and an F1 score are based on the GRU model's predictions, which are not particularly impressive. With the intention of comparing this model to others, the latter has high true positive rates of 3,081 (normal) and 1,371 (suicidal) states in its confusion matrix. Its accuracy is poor, however, when it is trying to differentiate more closely related disorders, such as anxiety vs. bipolar, where it is perhaps less clear where the confusion is coming from, as seen in Figure 13. Since there are misclassification rates in the reactions to classes, the GRU is not very effective at differentiating between the types of nuanced emotional expressions. This suggests that, although the GRU architecture is computationally fast and effective for simple categories of conditions, it may not be the most successful design for tasks that require a deep exploration of fine-grained health conditions.

#### 4.3.2 LSTM model

**Figure 14:**
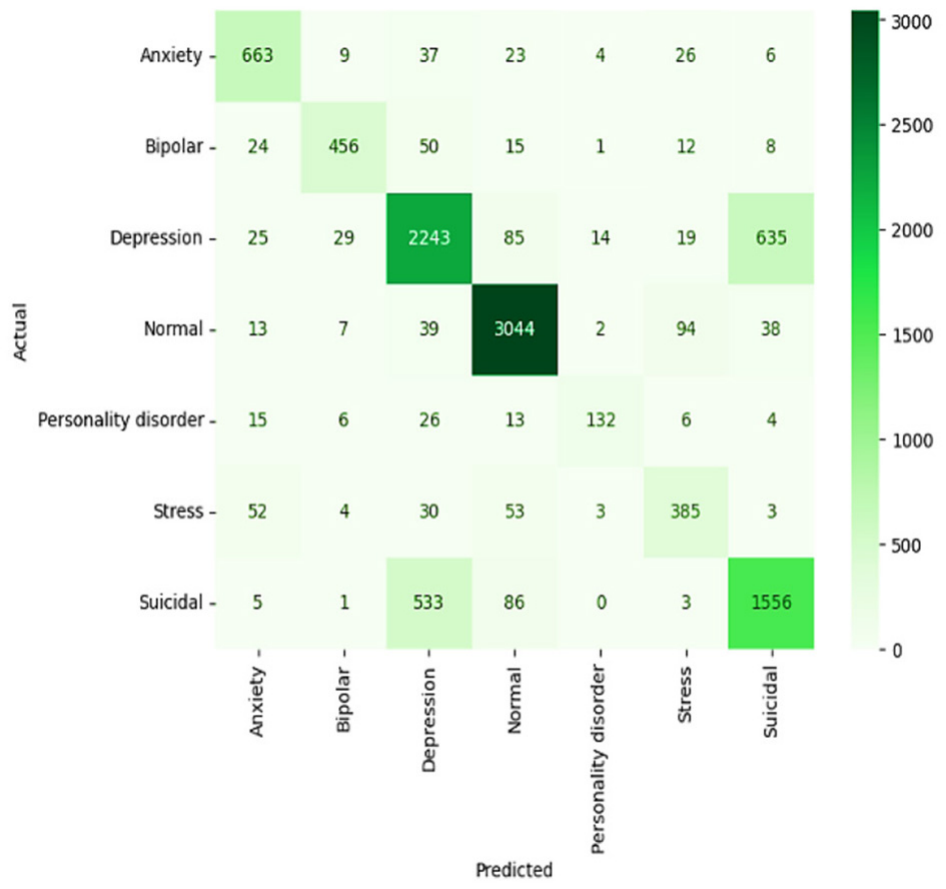
Confusion matrix of LSTM model.

It achieves an accuracy score of 79%, which outperforms the F1 score and reflects a deeper ability to model sequence-type data and their longer dependencies in this context. The LSTM confusion matrix shows strong potential for classifying normal and depression states. However, performance is hindered by the significant overlap between symptoms of depression and suicidal ideation—for example, 657 depression cases were misclassified as suicidal ideation, as illustrated in Figure 14. This issue highlights the challenge for reliable sentiment analysis, as the textual cues for sorrowful feelings and suicidal ideation are often closely related. Nevertheless, LSTM remains generally strong in handling a variety of mental health labels.

#### 4.3.3 Bi-LSTM model

**Figure 15:**
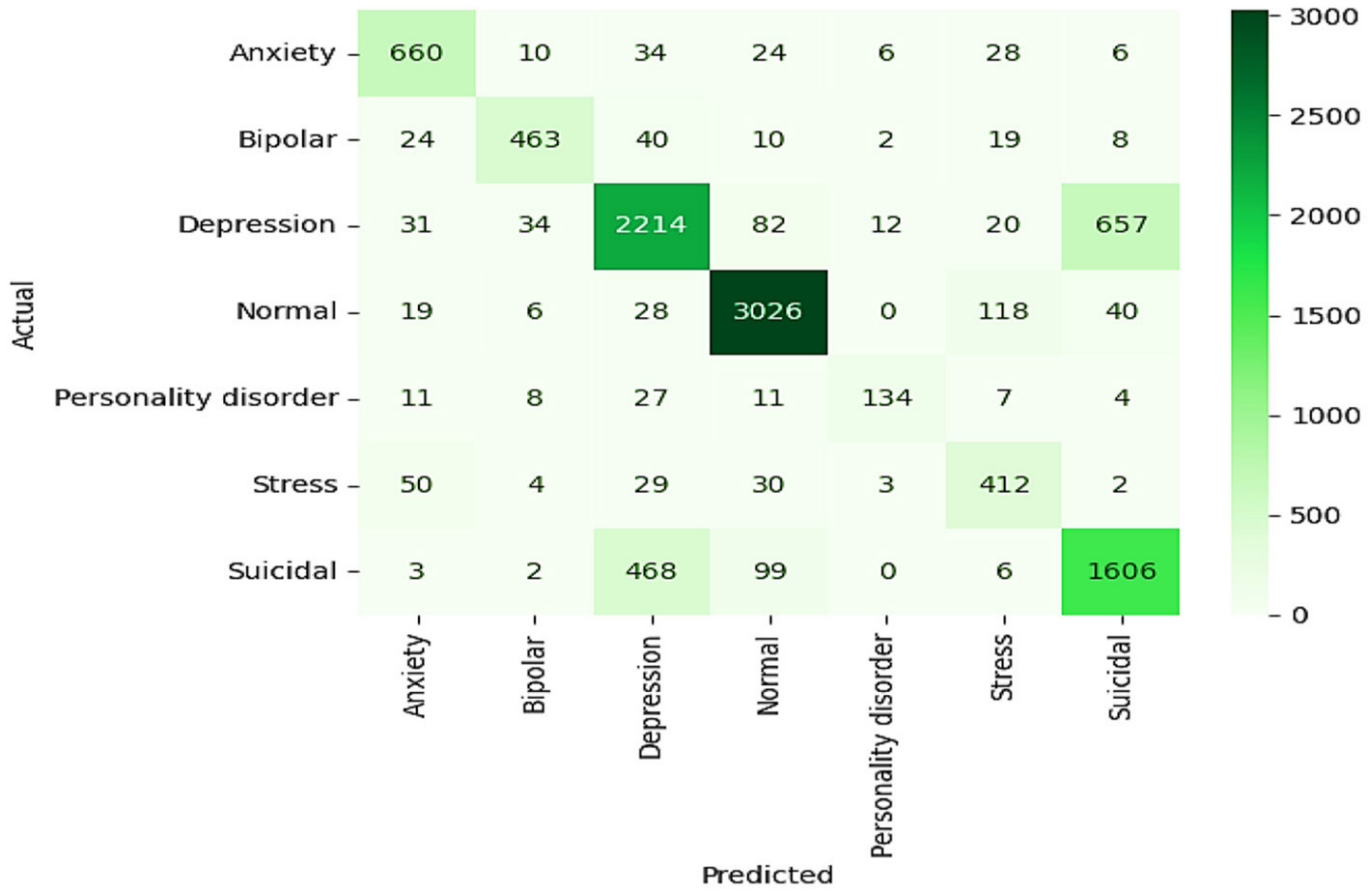
Confusion matrix of Bi-LSTM model.

The BiLSTM model exhibits the most robust performance, with an accuracy of 81% and an F1 score of 79%, and is therefore the most accurate among the three. It has a superbly well-distributed accuracy value in its confusion matrix, shown in Figure 15, with particularly good “normal” and “suicidal” state correct classification rates. As a result, the Bi-LSTM can process data from both past and future input sequences bidirectionally, offering a comprehensive understanding of the data. This allows for considerable confusion reduction compared to other models, especially in distinguishing overlapping symptoms in various conditions. Regarding predictive performance and more general network capabilities related to mental health analysis, with higher accuracy and sensitivity, the Bi-LSTM is regarded as the model of choice for mental health monitoring, particularly in critical applications.

The performance of the RoBERTa-LARGE and ELECTRA models demonstrates their strengths and differences when applied to the sentiment analysis of mental health conditions among students. As shown in Table 5, the RoBERTa-LARGE model achieves slightly higher accuracy and is robust enough to handle complex linguistic features and nuances, as evidenced by the significantly higher values of precision and recall compared to the state-of-the-art model. However, the training accuracy and validation accuracy do not align as well, suggesting that the model may be overfitting. In contrast, the ELECTRA model presents an overall score (accuracy, precision, recall, and F1 score) of 91% and less variation between the training and validation metrics (although with lower absolute performance). Finally, both models suggest confusion areas between closely similar mental health conditions such as bipolar and depression, which are a particular challenge to the sentiment analysis when fine emotional expressions need to be interpreted in a sophisticated way. Thus, ELECTRA may be advantageous over RoBERTa-Large in the real world, where generalizability across potentially heterogeneous data is important. Modeling different deep learning models in sentiment analysis for mental health classification, such as GRU, LSTM, BiLSTM, ELECTRA, and RoBERTa-Large, exhibits the ordering of their performance. At baseline performance levels, as measured by the GRU model, the accuracy is 77%. It is still a low percentage, but this is because the model is quite simple and lacks sufficient information to provide a comprehensive result on the emotional state of the situation. With LSTM and BiLSTM, there is a significant improvement in learning dependencies in sequence data, resulting in an F1 score of 79%. The ELECTRA model performs well on many metrics, achieving an overall accuracy of 91%, which is attributed to its transformer-based architecture that pushes contextual learning to the extreme. However, the best RoBERTa-Large model is the clear winner in terms of maximum performance, achieving 97% accuracy and a 94% F1 score, demonstrating its powerful capacity in capturing fine-grained language representations that describe language specificity related to mental health states. The sequence of these two developments recalls the significant impact that the arrival of advanced NLP technologies may have on improving the quality and credibility of mental health assessments based on text.

**Table 5:** Comparison of applied models (Results in %).

**Model**	**Accuracy**	**Precision**	**Recall**	**F1 score**	**Macro F1**	**Micro F1**
GRU	77	75	74	77	76	77
LSTM	79	79	78	79	78	79
BiLSTM	81	75	78	79	78	81
ELECTRA	91	91	91	91	91	91
RoBERTa-Large	97	95	91	94	93	97

In Table 5, the macro- and micro-average F1 scores allow for a relatively more balanced evaluation in the presence of class imbalance. It is true that traditional RNN-based models, such as GRU, LSTM, and BiLSTM, perform well with macro F1 scores ranging from 76 to 78%, but transformer-based approaches greatly improve this performance. ELECTRA is the most consistent across different metrics, but RoBERTa-Large is the best-performing among all baseline models, with a macro F1 score of 93% and a micro F1 score of 97%, indicating its stronger generalization to minority classes while retaining high overall accuracy.

**Figure 16:**
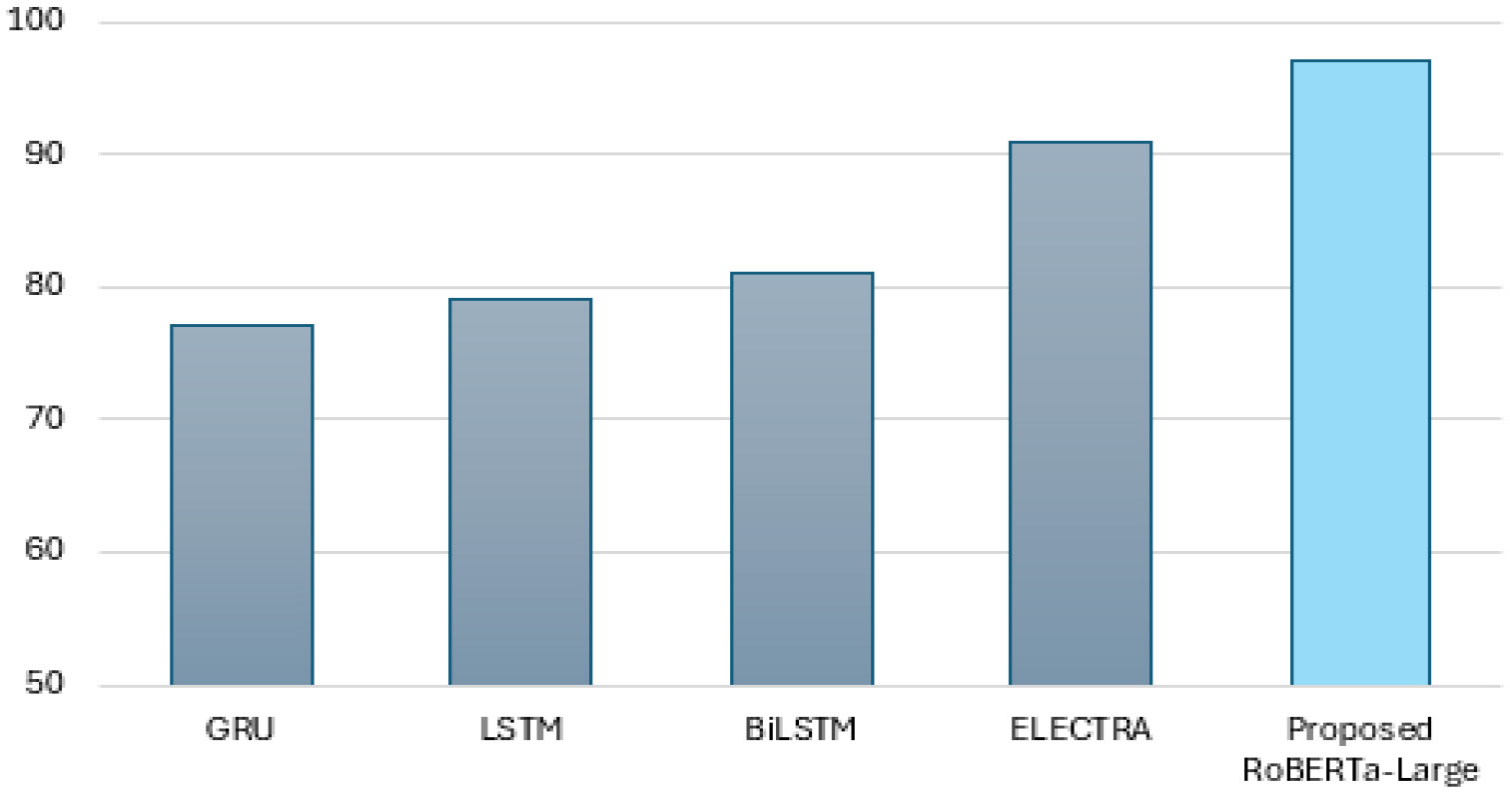
Applied model performance analysis.

This analysis strongly supports the use of advanced model architectures, such as RoBERTa-Large, for contextually sensitive and intertwined tasks, including analyzing text data to predict patients' mental health status, as depicted in Figure 16. These models provide gains in both predictive accuracy and reliability, qualities essential for applications where the precise meaning of emotional and psychological states must be assessed as accurately as possible.

### 4.4 Comparison with existing studies

Table 6 presents various feature-based models applied in mental health cases to yield results. A comparative analysis of the proposed RoBERTa-Large model with existing models shows that our model achieves a 97% result in sentiment analysis of mental health, relying on word embedding's. Indeed, this model surpasses other approaches, such as the traditional LSTM, which utilizes HRV from wearable devices, as well as textual features from Twitter, to achieve results of 83% and 74% in earlier applications. Among more advanced models, such as BiLSTM+BERT and other models like MentalBERT and RoBERTa+BERT, which perform well on complex text data and social media interactions, the highest performance achieved is only up to 89% and 79%, respectively. For example, a more recent development, XLNet, which utilizes word embedding's for social text-based communication, was more effective than RoBERTa-Large, achieving an outcome of 92%. Based on the above, the RoBERTa-Large model is selected for its superior performance, as well as its effective handling of word embedding's and robust training, which likely corresponds to a deeper, more nuanced understanding of language context for mental health. This makes it a brilliant tool in the domain of mental health diagnostics and sentiment analysis.

**Table 6:** Comparison with existing studies.

**Ref**	**Year**	**Model**	**Dataset**	**Results (%)**
Bokolo and Liu (2024)	2020	LSTM	HRV data	83
Coutts et al. (2020)	2021	LSTM	Twitter	74
Boer et al. (2021)	2022	Bi-LSTM	Social media	89
Chiong et al. (2021)	2023	MentalBERT	Facebook, Twitter	76
Vajre et al. (2021)	2024	RoBERTa,	textual data	79
Baek and Chung (2020)	2025	XLNet	Social media	92
Proposed		RoBERTa-Large	Sentiment analysis on mental health	97

## 5 Conclusion and future research

The role of activities in shaping students' physical and mental wellbeing is significant, which makes monitoring their mental health essential. Sentiment analysis, facilitated by advancements in AI, provides a powerful means to assess and understand the psychological states of sports students from the vast quantities of textual data they generate. The findings demonstrate that this technology provides valuable insights into the complexities of mental health patterns within this demographic. We have utilized a suite of AI-based models to capture subtle linguistic cues that reflect different mental health concerns. Of these models, our proposed RoBERTa-Large (96.5%) has performed impressively, achieving over 97% accuracy in the task of detecting and interpreting mental health-related sentiments. With its capability to process word embeddings and fine-tune training using big data, this model achieves the highest level of precision among existing models, making it an invaluable aid in addressing the mental health issues of students. This finding demonstrates the success of applying advanced AI models such as RoBERTa-Large in psychological health analysis. Moreover, it highlights the potential of AI models to revolutionize the way we perform analysis and treat people regarding mental health in educational environments. Going forward, there are numerous opportunities to monitor and improve students' mental health by utilizing AI-based sentiment analysis. Most importantly, combining other forms of data, including video, audio, and physiological measurements, with text analysis will enable a richer description of the student's mental state. These extra-modality data may represent non-verbal or physiological cues that are unavailable when using text only and could lead to a more precise and thorough assessment, ultimately increasing the reach of AI in mental health evaluations and efforts to incorporate these technologies into daily life, thereby improving support systems for students.

## Data Availability

The original contributions presented in the study are included in the article/supplementary material, further inquiries can be directed to the corresponding authors.
